# Editorial: New perspectives on the role of sensory feedback in speech production, volume III

**DOI:** 10.3389/fnhum.2026.1923499

**Published:** 2026-07-27

**Authors:** Jeffery A. Jones, John F. Houde

**Affiliations:** 1 Department of Psychology, Wilfrid Laurier University, Waterloo, ON, Canada; 2 Department of Otolaryngology - Head and Neck Surgery, University of California San Francisco, San Francisco, CA, United States

**Keywords:** auditory feedback, sensorimotor adaptation, sensory feedback, somatosensory feedback, speech motor control, speech production, sensory prediction error, multisensory integration

Research on sensory feedback in speech production has moved beyond the question of whether speakers use feedback. That question is largely settled. Speakers monitor the sensory consequences of their speech, compare them with expected outcomes, and use the discrepancies to adjust ongoing and future productions. Volume I focused largely on auditory feedback (Houde et al., 2023); Volume II established that somatosensory feedback is no less central (Houde et al., 2026); Volume III turns to how the speech feedback control system weights feedback errors, how it weights the different senses, and how that weighting is altered in disorder.

## Correction depends on more than the size of the error

Simple models of feedback control assume that the mismatch between predicted and actual feedback is estimated and the controller corrects errors in proportion to their size. The contributions in this volume complicate that account. Sreedhar and Daliri exposed speakers to formant perturbations that varied in direction, magnitude, and the extent of exposure. They found larger corrective responses when a perturbation moved a vowel towards a neighbouring natural vowel category. For these category-directed perturbations, responses to smaller shifts were proportionally largest early in exposure. These patterns are consistent with the speech motor control system weighting errors by their plausibility as self-generated, not simply by their magnitude. Error processing, in short, seems to hinge on what kind of error occurs and how often it recurs, and may also depend on whether the system treats it as self-caused rather than externally caused.

Gaines et al. challenged a common assumption: that a speaker who adapts to a specific perturbation will adapt the same way the next time they encounter it. When speakers heard the same 100-cent pitch perturbation across three repetitions within a single session, adaptation fell by 67% from the first repetition to the second and was effectively eliminated by the third, with neither differing significantly from baseline. This attenuation, the authors note, parallels implicit learning in visuomotor reaching tasks, reinforcing the characterization of pitch adaptation as primarily implicit and driven by sensory prediction error. This instability also raises practical concerns for studies that treat adaptation as a reliable individual-difference measure.

The physical effort of compensation has received little attention in the literature, but Ronayette et al. demonstrated that it limits sensorimotor adaptation. Speakers compensating for formant perturbations while holding a deformable lip tube that made lip rounding more effortful reached a plateau in lip-muscle activity at only about 56% of their maximum voluntary contraction, well below the physiological limit. This saturation suggests a comfort threshold rather than a biomechanical ceiling. When lip-rounding effort became too costly, some speakers shifted towards tongue backing, inferred from their formant trajectories. Partial adaptation, usually attributed to other factors, may also reflect effort optimization. Past a point, the larger the error, the more of it goes uncorrected, suggesting that further correction costs more effort than the system deems it worth.

## Feedback control is fundamentally multisensory

Several contributions in Volume III deepen Volume II's case for somatosensory feedback. Cummine et al. used fNIRS to compare oral stimulation (a lollipop) with oral anaesthesia (lidocaine) across two covert speech tasks. In the stored-articulation task, stimulation produced greater activity than anaesthesia in several regions, including the supramarginal and inferior frontal gyri. Given the established role of somatosensory feedback in overt speech, these findings suggest that speech-related somatosensory processing may remain engaged during covert production, even without overt movement.

Coleman et al. compared spontaneous rates across speaking, singing, tapped melody, and silent tapping. Individual speaking rates correlated with rates from singing and tapped-melody tasks, which produced sound, but not with silent tapping. Bayesian analyses supported both the positive correlations and the null correlations with silent tapping. How fast we speak, then, appears linked less to the vocal tract or speech domain than to whether the action produces sound.

Two contributions examine sensory feedback in second language acquisition. Suemitsu et al. trained Japanese speakers to produce American English /æ/ using real-time visual feedback of tongue position, without presenting native model sounds during the 10–15-min production training. Group productions moved closer to native acoustic targets, and discrimination accuracy subsequently increased for contrasts containing /æ/, whereas accuracy for the untrained /α/Ű/∧/ contrast showed no reliable change. This target-specific pattern is consistent with transfer from articulatory learning to perception and underscores the close coupling between speech production and perception. Li et al. found that for a novel second-language vowel, somatosensory acuity, indexed by a phonetic-awareness task (a measure of explicit articulatory awareness), was the only significant predictor of production accuracy, while auditory acuity was not. Together with the somatosensory findings from Volume II, these studies reinforce the view that feedback control in speech relies on multisensory information.

## Disorder reveals the architecture

The clinical contributions in this volume each probe a different parameter of the feedback control system by investigating what changes when it is compromised. Daliri et al. found that delayed auditory feedback increased trial-to-trial formant variability in typically fluent speakers but decreased it in adults who stutter. In stuttering, delayed feedback does not merely have a weaker effect; it reverses the effect, lowering variability rather than raising it. The system may respond more strongly to small, naturally occurring errors in these speakers, and more weakly to the large errors introduced by delaying feedback. What differs in stuttering may be how perceived errors are weighted.

Basciano et al. compared responses to gradual and sudden formant perturbations in individuals with aphasia. Under gradual perturbation, F1 corrective responses differed significantly from controls; under sudden perturbation, no adaptive or corrective group contrast was significant. The authors suggest that this schedule dependence may reflect altered auditory-error detection or error attribution. In single-case analyses of gradual F1 responses, both participants with qualitative apraxia-of-speech features showed adaptive-only impairments, consistent with possible feedforward deficits. Overall, responses under the sudden schedule more closely resembled controls, contrasting with limb rehabilitation studies after stroke, where gradual schedules have produced better outcomes.

Rangwala et al. extended the pitch centring paradigm featured in Volume II, where (Subrahmanya et al. 2024) examined centring in Alzheimer's disease, to laryngeal dystonia. Individuals with laryngeal dystonia began farther from the median pitch and made larger movements both towards it on centring trials and away from it on anticentring trials, although the proportions of these trial types did not differ between groups. The authors propose that elevated controller gain may amplify corrections enough to overshoot the median, whereas controller noise may produce misdirected anticentring. Vocal instability may therefore reflect pitch adjustments that are too strong or misdirected, consistent with a computational model of focal task-specific dystonia in which excessive sensorimotor-loop gain destabilizes motor output.

## Mapping the limits of feedback control

Read together, the contributions specify how the speech feedback control system works: how it weights feedback errors, how it weights the senses, and how that weighting breaks down. The clinical findings are not peripheral. They stress-test models built on typical speakers and, in each case, identify architectural features of the speech motor system that would otherwise have remained hidden.
